# Research progress on the role of FGF21 in insulin resistance

**DOI:** 10.3389/fendo.2025.1619462

**Published:** 2025-08-13

**Authors:** Haixin Zhang, Rongyu Zhu, Quanhao Sun, Likun Du

**Affiliations:** ^1^ School of Graduate Students, Heilongjiang University of Chinese Medicine, Harbin, Heilongjiang, China; ^2^ The Second Department of Endocrine Diseases, First Affiliated Hospital, Heilongjiang University of Chinese Medicine, Harbin, Heilongjiang, China

**Keywords:** insulin resistance, FGF21, signaling pathway, glucose, hyperinsulinemia

## Abstract

The elevated global prevalence of metabolic syndrome and type 2 diabetes mellitus (T2DM) has led to a dramatic increase in patients with insulin resistance. The majority of insulin resistance is closely associated with obesity and metabolic syndrome, resulting in impaired insulin signaling pathways. type 2 diabetes can be preceded by insulin resistance, and therefore, it is crucial to stop the progression of insulin resistance to type 2 diabetes. Fibroblast growth factor 21 (FGF21) holds a bright future in the therapeutic study of insulin resistance; it is involved in the regulation of lipid metabolism and immune homeostasis while ameliorating the impaired insulin signaling pathway, improving the state of insulin resistance through multiple aspects. In this review, we describe the physiological properties and signaling pathways of FGF21 and elaborate on the mechanism of action of FGF21 in improving insulin resistance. Finally, the progress of FGF21 analog research is summarized in the context of the treatment of insulin resistance.

## 1 Introduction

In recent years, the prevalence of Insulin Resistance (IR) has been increasing year by year worldwide. IR refers to the decreased or impaired sensitivity of target organs or target tissues to insulin, which is manifested as impaired glucose uptake and utilization, and this pathological response causes insulin secretion disorders, which elevate plasma insulin levels and lead to hyperinsulinemia. IR is attributed to several factors, including obesity, physical inactivity, advanced glycosylation end products (AGE), excess free fatty acids (FFAs), psychological stress, smoking, alcohol consumption, and certain medications. It is closely linked to obesity, metabolic syndrome, polycystic ovary syndrome, and other disorders, indicating that IR results from crosstalk alterations among various tissues (1, 2). When IR results in hyperinsulinemia, it is often associated with a diagnosis of impaired myocardial insulin signaling, mitochondrial dysfunction, and endoplasmic reticulum stress (3). Hyperinsulinemia is associated with elevated morbidity and mortality related to cardiovascular complications (4). IR may contribute to cancer development, as epidemiological studies indicate an elevated risk of breast, pancreatic, liver, and colorectal cancers in affected patients (5, 6). Adipose tissue plays a key role in the development of IR and may promote IR in other organs by releasing lipids and other circulating factors (7). IR can also appear as a pathologic manifestation in diseases such as early T2DM and cardiovascular disease.IR serves as the key core of pre-diabetes, and improving and treating insulin resistance can help reverse pre-glycemia. When insulin secretion is insufficient to counteract insulin resistance, then T2DM results. Meanwhile, central insulin resistance is thought to underlie cardiometabolic diseases due to the important role of insulin in brain circuits that control food intake and voluntary activity (8, 9). Furthermore, brain insulin resistance correlates with various cognitive deficits and significant neurodegenerative disorders, including Alzheimer’s disease and Parkinson’s disease (10). Currently, there is no specific treatment plan for IR, and the mechanism of insulin resistance is not yet fully understood.

Fibroblast growth factor (FGF) has multiple isoforms, and the FGF subfamily includes FGF19, FGF21, and FGF23. FGF21, a novel FGF expressed in the liver, is a signaling protein containing 208 amino acids, and FGF21 protects against injury caused by metabolic abnormalities (11, 12). FGF21 is an autocrine, paracrine, and endocrine factor synthesized by multiple organs with beneficial effects on weight loss and glycemic improvement, acting on multiple target tissues to increase fat utilization and energy expenditure, improve glucose homeostasis, and increase insulin sensitivity (13). Thus, FGF21 plays an important role in regulating glucose and lipid homeostasis (14, 15). FGF21 expression and levels are influenced by diet, nutritional status, hormones, and activity of related transcription factors (16–18).FGF21 has been shown to have a positive role in ameliorating insulin resistance and to mediate glucagon action (19, 20). This review summarizes the studies of recent years, detailing recent advances in the study of FGF21 in insulin resistance, and develops the new potential of FGF21 for the treatment of IR.

## 2 Mechanisms of insulin resistance

The occurrence of IR is a complex process, and its mechanism has not been fully clarified so far. However, some studies have confirmed that IR is mainly caused by abnormal quality of β-cell function, abnormal insulin signaling pathway, inflammation, lipotoxicity, oxidative stress, and environmental and genetic factors. In a state of insulin resistance, β-cell depletion to maintain normoglycemia and compensate for insulin demand is critical to the pathogenesis of the condition (21). Simultaneously, lipotoxicity and elevated free fatty acids (FFA) overstimulate glucose-mediated insulin production in β-cells, enhancing β-cell signaling and oxidative stress, which ultimately leads to metabolic depletion of β-cells (22, 23). Glucose increases IRS2 expression in β-cells in response to elevated FFA levels as a compensatory mechanism for insulin resistance, thereby enhancing β-cell mass, which correlates with pancreatic β-cell neogenesis, proliferation, and survival (1). This elevates blood insulin levels, potentially leading to beta-cell damage due to hyperinsulinemia (24). Recent studies have found that increased expression of the receptor for advanced glycosylation end products (RAGE) correlates with inflammation, toxicity, and apoptosis in human IAPP-induced (h-IAPP-induced) beta cells and pancreatic islets. This is because RAGE triggers oxidative stress, inflammation, and apoptosis by binding to IAPP toxic intermediates (25). Additionally, β-cell death and disease progression are closely associated with heightened cellular glucose metabolism, the accumulation of saturated-chain fatty acid signaling, impaired insulinogen processing, abnormal insulin secretion, reduced β-cell mass, and pancreatic islet amyloid deposition in states of insulin resistance (26).

Insulin receptor substrates (IRS) are important nodes of insulin signaling and are closely related to insulin sensitivity. IRS plays an important role in the insulin signaling pathway. The IRS family includes IRS1 and IRS2, which are key proteins in insulin receptor and intracellular signaling (27, 28). Dysregulation of IRS pathways for insulin signaling is one of the common causes of IR. FFAs, inflammatory cytokines, and hyperinsulinemia significantly increase a variety of serine kinases such as IκB kinase (IKK), c-JunN-terminal kinase (JNK), specific isoforms of protein kinase C (PKC), and double-stranded RNA-dependent protein kinase (PKR) (29), and these kinases impede IRS1 function and can promote insulin resistance by promoting the expression of genes involved in activating inflammation and nuclear factor κB (NF-κB).

Therefore, the treatment of insulin resistance should be based on insulin signaling, lipid metabolism, glucose metabolism, oxidative stress, and inflammation. FGF21 has a good effect in improving glucose-lipid metabolism, insulin signaling, inflammation, and oxidative stress.

The pathogenesis of insulin resistance (IR) involves a complex interplay of β-cell dysfunction, lipid overload, chronic inflammation, and oxidative stress, all of which converge to disrupt insulin signaling. Key findings highlight that β-cell failure is both a consequence and driver of IR, as elevated free fatty acids (FFAs) induce lipotoxicity, hyperinsulinemia, and oxidative damage, ultimately depleting β-cell mass (21–26). Simultaneously, serine phosphorylation of IRS1/2 by stress kinases (e.g., JNK, IKK) exacerbates insulin signaling defects, creating a vicious cycle of metabolic deterioration (27–29).

FGF21 emerges as a promising therapeutic agent due to its pleiotropic effects—enhancing insulin sensitivity, suppressing lipotoxicity, and reducing inflammation—making it a potential multi-target intervention to break this cycle. Future research should focus on optimizing FGF21 analogs to maximize these benefits while minimizing species-specific limitations observed in clinical translation.

## 3 FGF21: biological characteristics

### 3.1 Gene expression and synthetic secretion of FGF21

FGF21 is a peptide hormone, and in rodents and humans FGF21 is secreted primarily in the liver to produce (30, 31), FGF21 is synthesized in minimal quantities by adipose tissue and can exert either autocrine or paracrine effects (32–34), FGF21 mRNA was detected in adipose, muscle, and pancreatic tissues (35, 36). Therefore, FGF21 is also known as a hepatic factor, adipokine, and myokine. Meanwhile, cold exposure,low-protein diet, exercise, fasting, and high fructose intake all led to an increase in FGF21 secretion (13, 37–41), but cold exposure only stimulated localized production of FGF21 in adipocytes, with no change in systemic FGF21 levels (42). FGF21 levels are increased in pancreatic islets and hepatocytes after higher glucose and fatty acid concentrations (43–45). Fasting and starvation for 6–12 hours resulted in an increase in circulating FGF21 in mice (46), but in humans, serum FGF21 was essentially unchanged after two days of fasting, and serum FGF21 was not significantly elevated until after seven days of fasting (47, 48). Notably, a three-month ketogenic diet leads to a decrease in circulating levels of FGF21 in humans (49). Growth hormone has been shown to promote FGF21 expression in mice, but not in humans, indicating a major divergence in the physiological regulatory role and expression of FGF21 between species (50, 51).

It was found that animals deficient in peroxisome proliferator-activated receptor α (PPARα) resulted in reduced FGF21 secretion under starvation (52). The FGF21 promoter can directly bind to PPARα for transcriptional activity (46, 52). 3T3-L1 adipocytes or human adipocytes sourced from subcutaneous adipose tissue are activated with the PPARγ agonist rosiglitazone, which concurrently elevates FGF21 mRNA expression (53). Evidence substantiates that the therapeutic impacts of PPARα agonists on lipid metabolism, as well as the hypoglycemic and insulin-sensitizing actions of PPARγ agonists, are facilitated by FGF21 (54–56). Consequently, it is further shown that FGF21 is a downstream target of PPARα and PPARγ, both of which are integral to FGF21 signaling and function.

The level of FGF21 secretion is also closely related to nutritional status and exercise, and sugar is a potent stimulator of FGF21. Oral administration of glucose or fructose, or intravenous injection, significantly increases circulating levels of FGF21, except that the two modes of ingestion lead to differences in the time point of FGF21 elevation (39, 57–61). A high-carbohydrate (HC) diet increases FGF21 mRNA and circulating FGF21 levels in mouse liver and muscle (62). Carbohydrates stimulate FGF21 gene expression through the reverse transcription factor carbohydrate response element binding protein (ChREBP). In addition, a low-protein diet leads to elevated circulating levels of FGF21 (63). Notably, the increase in FGF21 levels induced by a high-carbohydrate diet and a low-protein diet is regulated independently of each other (60). A low-protein diet results in a deficiency of essential amino acids, which impairs the regulation of the β2-adrenergic receptor signaling pathway by protein kinases in the liver and anterior pyriform cortex. This deficiency leads to elevated phosphorylation levels of eukaryotic initiation factor-2α (eIF2α) and upregulation of FGF21 transcription via the activation of transcription factor 4 (ATF4) (64). At the same time, exercise leads to elevated FGF21 levels. Resistance exercise (RE) elevates FGF21 levels higher than endurance exercise (EE) (65), and high-intensity exercise increases FGF21 levels more significantly than moderate-intensity exercise (66). Acute endurance exercise, however, induces the production of glucagon and FGF21, hence modulating the glucagon-FGF21 axis (67, 68).

### 3.2 FGF21 signaling and pathways

FGF21 exerts its effects by attaching to a receptor complex composed of the fibroblast growth factor receptor (FGFR) and the co-receptor β-klotho (KLB) at the amino and carboxy termini, respectively (69), and the co-receptor KLB is crucial for the activity of FGF21 (70). Meanwhile, the residues 198–200 at the carboxy-terminal of FGF21 are flexible and easily cleaved by proteolysis, eliminating the binding of FGF21 to the extracellular site of KLB, thus playing an important role in terminating FGF21 signal transduction (71).FGFR is a receptor tyrosine kinase family composed of FGFR1, FGFR2, FGFR3, and FGFR4. FGF21 has the highest affinity for FGFR1 and can signal through FGFR2 and FGFR3, but does not respond to FGFR4 (72). Although FGFR is widely expressed in many tissues, KLB expression is limited to certain metabolic tissues such as the pancreas, liver, adipose tissue, and brain (73, 74).KLB is a single-pass transmembrane protein with a unique domain that mediates signal transduction by binding to ligands through its extracellular domain (75).

FGF21 signaling pathways are complex and associative. The downstream pathways of FGF21 include AMPK, mTOR, HPA axis, GLUT1, lipocalin, etc., and are capable of indirectly affecting the signaling pathways of MAPK, JNK, and NF-κB. First, FGF21 can directly promote the expression of glucose transporter protein 1 (GLUT1) to increase glucose uptake. Meanwhile, FGF21 was able to promote lipocalin secretion to increase insulin sensitivity in adipose tissue and regulate lipid metabolism. Secondly, FGF21 controls energy metabolism by promoting the activation of the AMPK signaling pathway and regulating the HPA axis through the central system. activation of AMPK signaling indirectly increases the expression of GLUT4 to increase glucose uptake and improves IR, and FGF21 inhibits mTOR signaling to ameliorate insulin resistance due to impaired insulin signaling. FGF21 attenuates the effects of immunoinflammation on the insulin signaling pathway by inhibiting the immune inflammatory response caused by the activation of pro-inflammatory kinases such as JNK and IKK, as well as the activation of the NF-κB signaling pathway.

Adenosine monophosphate-activated protein kinase (AMPK) is closely related to the body’s energy metabolism. It governs energy metabolism by promoting the catabolism of glucose and lipids (76, 77). FGF21 appears to have a comparable function, suggesting that both may operate via the same pathway, or that the diverse metabolic effects of FGF21 are mediated through AMPK signaling. AMPK is recognized as a long-lived protein, and when FGF21 promotes AMPK activation, a series of metabolic responses occur. It has been found that FGF21 promotes AMPK signaling in several tissues, activating hepatic kinase B1 (LKB1) through FGFR1/β-klotho signaling or indirectly by stimulating the secretion of lipocalin and corticosteroids in adipose tissue to achieve activation of AMPK signaling in target tissues and to achieve control of energy metabolism, such as enhancing mitochondrial biogenesis (78–82). For example, FGF21 promotes the secretion of lipocalin, which stimulates lipocalin receptors (AdipoR1/2) and triggers AMPK activation through APPL1/LKB1 signaling. FGF21 also mediates the activation of AMPK signaling by corticosteroids through the HPA axis via CB1 and or GR receptors (83). FGF21-induced AMPK activation was not observed in differentiated human subcutaneous adipocytes, indicating that FGF21’s effect on AMPK may be tissue-specific (84). AMPK can upregulate GLUT4 expression by phosphorylating the transcriptional repressor histone deacetylase (HDAC) 5, phosphorylates glucose transporter protein targets to promote glucose utilization and cellular uptake, and also inhibiting fatty acid, cholesterol, and protein synthesis (85–88). This was also confirmed in animal research experiments, where AMPK activators improved diabetic symptoms, including in rodents, and reduced blood glucose levels in a T2DM model by promoting muscle glucose uptake (89, 90).

FGF21 activates the AMPK-SIRT1 signaling pathway in adipocytes, and AMPK enhances peroxisome proliferator-activated receptor gamma coactivator 1α (PGC-1α) expression by inducing the expression of the cellular energy sensor Sirtuin1 (SIRT1), which promotes mitochondrial respiration and upregulates GLUT4 expression in muscle cells, resulting in a serum glucose level that is significantly reduced (91, 92). In experiments involving berberine, AMPK signaling enhanced PGC-1α expression and facilitated the reversal of insulin resistance, indicating the significant role of PGC-1α in addressing this condition (93). Moreover, AMPK inhibits gluconeogenesis through the phosphorylation of coactivators involved in the cAMP response element binding protein (CREB) and forkhead box protein O (FOXO) pathways (88). It has been found that insulin resistance in muscle and adipose tissue is closely associated with reduced AMPK activity (94–96). There exists a significant relationship between insulin resistance and the dysregulation of AMPK signaling (97). Excessive activation of AMPK signaling may be indirectly associated with the onset of neurological diseases, including Alzheimer’s disease. Excessive stimulation of AMPK signaling may trigger autophagic cell death rather than facilitating survival-promoting autophagy (93).In addition, AMPK regulates the mammalian target of the rapamycin protein complex (mTORC1) to control cell growth and protein translation (88).

There is substantial research evidence that FGF21 promotes glucose assimilation by blocking the mTORC1 molecule, thereby increasing insulin sensitivity in the liver, bone, muscle, and adipose tissue (98). The mammalian target of rapamycin (mTOR) signaling is involved in the regulation of pancreatic β-cell growth and proliferation and insulin secretion, thereby affecting glucose homeostasis (99, 100). mTOR coordinates peripheral insulin target tissues with the central nervous system to regulate food intake and glucose homeostasis, among other functions. Islets derived from individuals with type 2 diabetes frequently exhibit heightened mTORC1 activity alongside reduced mTORC2 activity (101, 102), indicating that increased mTORC1 activity may compromise β-cell functionality (103). Dysregulation of mTOR signaling results in abnormalities in glucose and lipid metabolism, as well as insulin resistance. Chronic activation of mTORC1 signaling is observed in individuals with overnutrition, type 2 diabetes mellitus (T2DM), and obesity (104, 105). FGF21 enhanced insulin signaling in mouse skeletal muscle by inhibiting mTORC1, which in turn reduced IRS1 phosphorylation at Ser636/639 and improved insulin sensitivity (106).

FGF21 inhibits the activation of pro-inflammatory kinases such as JNK and PKC, thereby inhibiting NF-κB signaling. NF-κB is a key node connecting inflammation, oxidative stress, and insulin signaling abnormality, and plays an important regulatory role in the development of IR. The nuclear factor kappa-light-chain-enhancer of activated B cells (NF-κB) typically exists in an inactive form. Upon stimulation by proinflammatory kinases, the endogenous inhibitor IκB undergoes phosphorylation, resulting in its dissociation from the NF-κB-IκB complex. This dissociation leads to the ubiquitination and degradation of IκB, facilitating the translocation of NF-κB into the nucleus, where it binds to the κB site in the promoter region to activate target genes (107). The activation of NF-κB increases the expression of proinflammatory factors that trigger Oxidative stress and inflammatory responses, and inflammation and oxidative stress increase serine and threonine phosphorylation of IRS1 and decrease tyrosine phosphorylation to inhibit IRS1 activity, leading to impaired insulin signaling (108, 109).

In addition, glucagon also plays a role in the FGF21 pathway. It has been found that deletion of the glucagon receptor prevents the elevation of FGF21 during starvation. If acute administration of glucagon in animals leads to an increase in circulating FGF21 levels, these findings suggest that FGF21 secretion is closely related to, and may be regulated by, glucagon (110), and the control of FGF21 by glucagon is independent of circulating insulin and unrelated to insulin levels (111). Glucagon and glucagon agonists enhance FGF21 mRNA expression in the liver and elevate circulatory FGF21 concentrations (67, 111). Glucagon, as an antagonist of insulin, plays an important role in increasing hepatic glucose production during starvation, whereas FGF21 inhibits hepatic glucose release; therefore, glucagon may be an important mediator of FGF21 signaling and function. Glucagon promotes activation of the cAMP and protein kinase A (PKA) pathways, and glucagon’s promotion of PKA activation leads to an increase in FGF21 secretion with no change in FGF21 mRNA levels (112). It has been found that glucagon activates the AMPK signaling pathway and promotes the expression of PPARα, which in turn promotes the expression of FGF21 (110).In mice lacking the glucagon receptor, glucagon, on the other hand, failed to induce PPARα and FGF21 gene expression and hepatic AMPK phosphorylation (113). It was found that chronic GcgR agonist-induced energy expenditure mainly originated from increased sympathetic nerve excitation and led to BAT tissue thermogenesis and WAT browning (114), which was similar to the effect of FGF21 action. Meanwhile, the regulation of glucagon receptors on glucose metabolism, lipid metabolism, and body weight adiposity was also affected by FGF21 (115), it also indicates that FGF21 interacts with and affects glucagon.

In summary, FGF21 can improve insulin resistance by improving insulin signaling and affecting its downstream pathway. When the insulin signaling pathway is impaired, FGF21 enhances insulin signaling by activating the AMPK signaling pathway and inhibiting the mTOR signaling pathway to achieve glucose uptake and improve insulin resistance. FGF21 regulates lipid metabolism by enhancing lipocalin secretion and promoting thermogenesis in brown adipose tissue (BAT) while facilitating the browning of white adipose tissue (WAT). This process increases glucose uptake by adipose tissue and improves IR.

**Figure 1:**
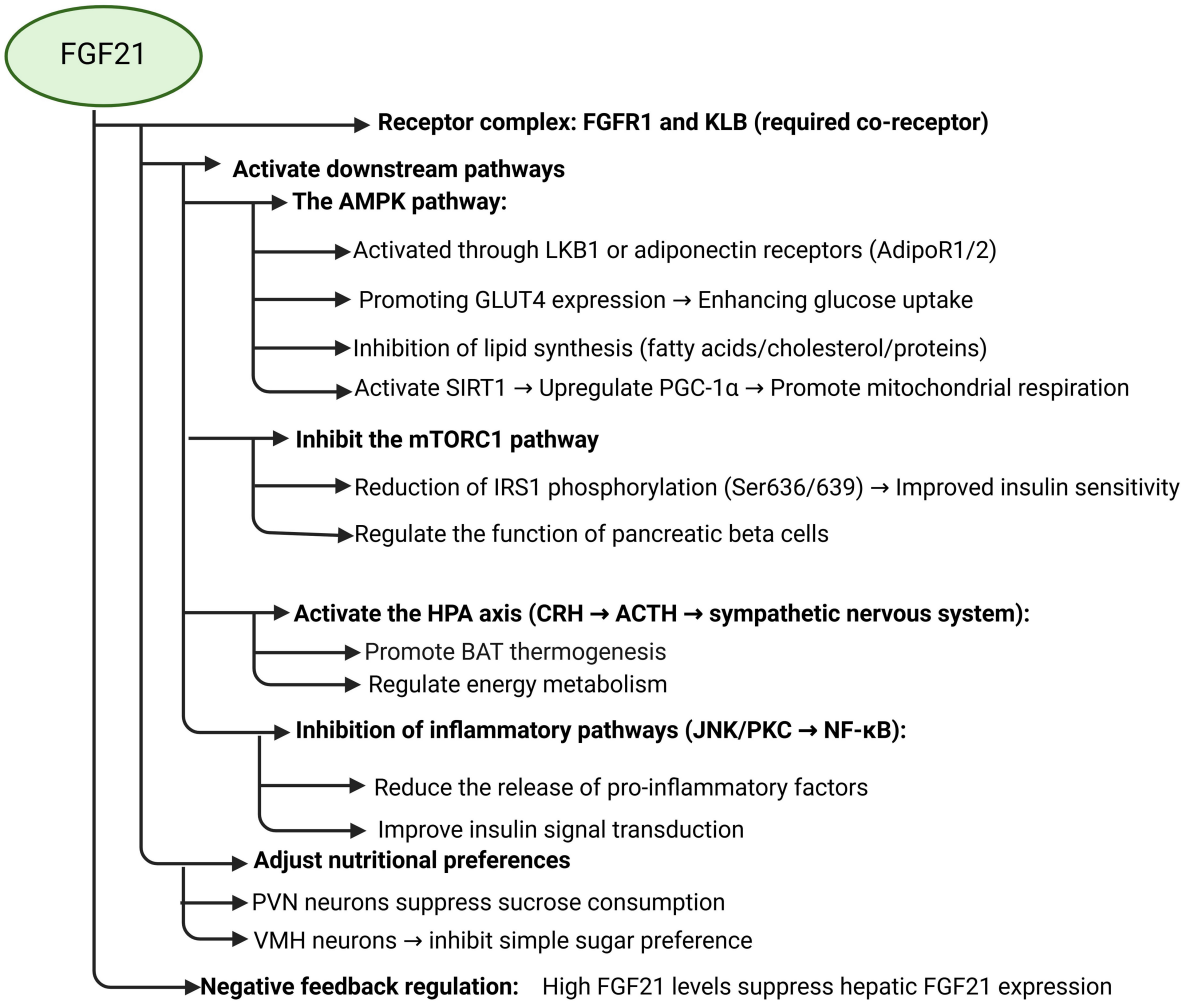
Schematic diagram of FGF21 and its related signaling pathways.

FGF21 emerges as a multifunctional metabolic regulator shaped by nutritional, hormonal, and environmental cues. Its secretion is tightly controlled by fasting, macronutrient intake (carbohydrates/proteins), and exercise, with tissue-specific responses (e.g., adipose-limited cold exposure) highlighting its systemic yet context-dependent roles. As a downstream effector of PPARα/γ, FGF21 links lipid/glucose metabolism to transcriptional networks governing energy homeostasis (52–56). Mechanistically, FGF21 integrates AMPK activation, mTORC1 inhibition, and anti-inflammatory pathways to enhance insulin sensitivity, promote lipid oxidation, and restore metabolic balance (**Figure 1**). Notably, its therapeutic potential is underscored by tissue-specific effects (e.g., adipose AMPK activation) and interactions with glucagon signaling, though species differences in regulation (e.g., growth hormone responsiveness) warrant further investigation (50, 51, 83, 84). These findings position FGF21 as a promising target for metabolic disorders, provided challenges like variable pharmacokinetics and off-target effects are addressed.

## 4 FGF21 and insulin resistance

### 4.1 The role of FGF21 in the central nervous system

The central nervous system (CNS) plays a critical role in mediating FGF21’s metabolic effects, including dietary preference regulation, circadian rhythm modulation, thermogenesis enhancement, hepatic insulin sensitivity improvement, and lipid metabolism control (42, 116–119). CNS-specific FGF21 knockout mice exhibit abolished body weight and glucose regulation effects (116), while FGF21 receptors and co-receptors (KLB) are expressed in key hypothalamic nuclei (NTS, SCN, PVN) (19, 117), confirming the CNS as a direct target organ. Notably, FGF21 is detectable in human cerebrospinal fluid (CSF), with levels correlating directly with serum concentrations (118), and can cross the blood-brain barrier after hepatic secretion (119).

FGF21 exerts specific effects on dietary preferences through distinct hypothalamic pathways (30, 58, 63, 74, 120–122). It reduces sucrose intake by activating PVN neurons while suppressing simple sugar consumption via VMH signaling (74). FGF21 transgenic mice show selective reductions in sugar/artificial sweetener preference without affecting other nutrients (58). This regulation occurs independently of taste perception (121), as evidenced by: (1) 62% reduction in high-sugar diet intake following PVN-targeted FGF21 administration (58), and (2) abolished dietary preference modulation in brain-specific KLB knockout mice (58, 122). Importantly, these effects are reversible and exhibit transient PVN dependency (58).

In addition to dietary regulation, CNS FGF21 signaling significantly impacts energy metabolism (123). Through FGFR1/KLB activation, FGF21 stimulates CRH expression in the SCN via ERK1/2-CREB pathway (19), subsequently increasing ACTH secretion and sympathetic nerve activity in brown adipose tissue (BAT) (124). This cascade enhances adipose browning and thermogenesis. Notably, CRH antagonists block FGF21-induced sympathetic activation, while glucocorticoids may indirectly impair hepatic insulin sensitivity by suppressing osteocalcin production (124).

A negative feedback mechanism governs FGF21 regulation (125, 126). High-dose FGF21 administration suppresses hepatic FGF21 gene expression more potently than streptozotocin treatment alone, with similar observations in independent studies, indicating that circulating FGF21 levels regulate its own gene expression (125, 126).

### 4.2 The role of FGF21 in glucose metabolism

FGF21 has demonstrated the ability to modulate blood glucose and insulin resistance in mice, effectively reducing lipid levels, blood glucose, and serum insulin, while enhancing insulin sensitivity in diet-induced obesity models, mostly through improved glucose absorption by skeletal muscle and diminished hepatic glucose synthesis (127).FGF21 can affect glucose homeostasis in both insulin-dependent and insulin-independent manners. Myogenic FGF21 positively regulates skeletal muscle insulin signaling to maintain systemic glucose stabilization and energy homeostasis (66, 73), including increasing glycogen conversion and KLB expression, etc. FGF21’s capacity to diminish the mRNA expression of glucose-6-phosphatase while leaving phosphoenolpyruvate carboxykinase unaffected indicates that FGF21 lowers glucose release from glycogenolysis (127). Increased hepatic glycogen, inhibition of hepatic gluconeogenesis, decreased glucagon, and improved glucose clearance were found in ob/+ mice when FGF21 was given acutely (128), and the overexpression of FGF21 in various transgenic mice improved glucose clearance, augmented insulin sensitivity, and reduced fasting blood glucose levels (129). But not in ob/ob mice. Moreover, FGF21 enhances insulin-stimulated glucose uptake in skeletal muscle and glucose uptake by 3T3-L1 adipocytes (130), stimulates insulin-independent expression of GLUT1, and increases hepatic glycogen synthesis (123, 131–136).

Furthermore, FGF21 can directly signal to brown adipose tissue to enhance insulin sensitivity and glucose uptake. FGF21 is synthesized and released in both white adipose tissue (WAT) and brown adipose tissue (BAT). Owing to a positive feedback mechanism, FGF21 is crucial in facilitating the metabolic advantages of PPARγ on glucose homeostasis and peripheral insulin sensitivity (55).In humans and rodents, BAT is extremely insulin-sensitive, with the ability to uptake glucose and generate heat (137, 138), and possesses a comparable capacity to metabolize glucose and enhance insulin sensitivity as skeletal muscle (136).In contrast, in WAT, FGF21 stimulates glucose uptake, regulates lipolysis, and enhances PPARγ activity in an insulin-independent manner (50, 55, 139). Intraperitoneal administration of FGF21 in ad libitum-fed wild-type mice did not alter plasma glucose concentrations; however, co-administration of FGF21 with insulin produced a synergistic effect on plasma glucose, surpassing the impact of insulin alone (140), indicating that FGF21 may augment insulin sensitivity and amplify insulin action. FGF21 was found not to affect enhancing insulin sensitivity in starved mice devoid of adipose tissue (141). This reduction in blood glucose, attributed to improved insulin sensitivity, primarily relies on the augmented peripheral glucose disposal by BAT (140, 142, 143), while not elevating glucose uptake in WAT. Likewise, the hypoglycemic impact of FGF21 is entirely abolished when KLB is removed from adipocytes, although it remains intact when KLB is removed from the liver (123, 140). The lack of KLB in mouse thermogenic (UCP1+) adipocytes compromised the enhanced insulin sensitivity of FGF21 (11). These data confirm the significant involvement of FGF21 in adipocytes in decreasing peripheral glucose levels by improving insulin sensitivity.

FGF21 promotes the expression and secretion of lipocalin through PPARγ, and there is a large expression of adipoR1 and adipoR2 in skeletal muscle, heart, liver, and other organs (144). Lipocalin enhances glucose homeostasis and increases insulin sensitivity in adipose and muscle tissues (145), indicating that the FGF21-lipocalin axis plays a synergistic role in the regulation of glucose metabolism. In leptin-deficient (ob/ob) mice and diet-induced obese mice, the pharmacological effects of FGF21 on glucose, dyslipidemia, and IR are reduced (72). Lipocalin secretion was significantly increased in mice overexpressing the FGF21 gene (129), whereas FGFR1 mutant mice exhibited no increase in lipocalin secretion following FGF21 treatment (146). However, a high-fat diet leads to impairment of the FGF21-lipocalin axis, which is ameliorated by prolonged exercise (120). Therefore, when FGF21 is used to treat patients with high-fat diet-induced insulin resistance, it should be combined with exercise therapy to repair the FGF21-lipocalin axis and restore their metabolic functions.

In conclusion, FGF21 demonstrates an irreplaceable role in insulin resistance, both by increasing insulin signaling to enhance glucose uptake and by increasing lipocalin secretion to elevate insulin sensitivity. FGF21 improves glucose metabolism not only through insulin-dependent but also non-insulin-dependent modes, which suggests that FGF21 plays an indispensable role in the process of glucose sugar metabolism, suggesting that FGF21 plays an indispensable role.

### 4.3 The role of FGF21 in lipid metabolism

The FGF21 receptor is highly expressed in adipose tissue, making adipose tissue an important target tissue for FGF21. FGF21 promotes lipolysis in isolated adipocytes, increases circulating free fatty acids, and enhances ketogenesis in the liver (46, 111). During starvation, FGF21 enhances hepatic gluconeogenesis, promotes fat oxidation, and stimulates ketogenesis (147). FGF21 transgenic mice exhibited an increase in hepatic fat β-oxidation, which serves as a crucial substrate for gluconeogenesis and ketogenesis. FGF21’s effects on lipid metabolism are also insulin-independent, as shown in type 1 diabetic and insulin-deficient animal models (111). In obesity, FGFR1 expression is reduced in adipose tissue (148). When adipocyte-specific FGFR1 was knocked down in mice, the effects of FGF21 in lowering plasma glucose, triglycerides, insulin and increasing energy expenditure were mostly eliminated (146). FGF21 and its analogs were shown to decrease the expression of adipogenic genes in DIO mice, including SCD1, FASN, and/or SREBF1, a key regulator of the adipogenic transcriptional network that is upregulated in response to a high-fat diet (127, 149, 150). Prolonged administration of FGF21 or FGF21 analogs in rodents and non-primates resulted in significant weight loss, with lesser effects on human body weight (140, 142, 151). In obese animals, the treatment with the medication FGF21 enhanced energy expenditure and ameliorated diabetes and obesity (142, 146). In DIO mice, prolonged administration of FGF21 led to a reduction in body weight along with a reduction in adiposity, hepatic triglycerides, and cholesterol (152). Thus, FGF21 also prevents hepatic steatosis and atherosclerosis (153).

FGF21 can directly promote glucose absorption and lipocalin release in adipocytes. The triglyceride-lowering effect was lost after the knockdown of lipocalin in DIO mice (145), indicating that FGF21 may function in a lipocalin-dependent way. FGF21 can increase the secretion of lipocalin, which plays an important role not only in glucose metabolism but also has an important influence on lipid metabolism. For example, FGF21 analogs can significantly increase plasma lipocalin levels in obese and type 2 diabetic patients (154). Pharmacologic dosages of FGF21 can stimulate lipocalin production in WAT (145, 155). The elevation in energy expenditure caused by FGF21 treatment was diminished in lipocalin-deficient animals. Exercise mitigated the reduction in FGF21’s capacity to stimulate lipocalin secretion produced by a high-fat diet, potentially attributable to enhanced expression of FGFR and KLB (120, 156). Lipocalin reduces the accumulation of lipids, such as ceramide, in insulin-sensitive tissues, whereas the presence of ceramide accumulation in the liver leads to IR and lipotoxicity.

FGF21 increases energy expenditure and decreases WAT content by activating BAT (157, 158). FGF21 knockout mice exhibit increased visceral and subcutaneous fat accumulation, along with enlarged adipocytes. In mice lacking FGF21 receptors, exercise enhances lipolysis and reduces WAT size, yet results in atypical lipid accumulation in non-adipose tissues, including the liver and muscle (119). This indicates that FGF21 inhibits the transfer of exercise-induced catabolic adipose tissue to non-adipose tissues, including the liver and muscle (156). Elevated FGF21 levels markedly decrease WAT size. The browning of WAT results in reduced adipocyte size and enhanced thermogenesis, correlating with the effects of FGF21-promoted genes (UCP-1, CIDEA, DIO2) on heat production. FGF21 is associated with the promotion of thermogenic gene expression, including UCP-1, CIDEA, and DIO2 (33, 159). UCP1 is not essential for the increases in energy expenditure mediated by FGF21 (160). When UCP1 is absent, energy expenditure is maintained by compensating for adipose tissue thermogenesis. Meanwhile, exercise increases mitochondrial citrate synthase activity, which is reduced in FGF21 knockout mice, suggesting that FGF21 regulates mitochondrial activity in WAT, and is likely to be involved in the regulation of WAT “browning” (161, 162). FGF21 exerts both acute and chronic influences on metabolic processes. Lipodystrophic mice exhibiting diminished adipose tissue demonstrate resistance to both the acute and chronic effects of systemic FGF21, a factor recognized for its ability to reduce blood glucose levels and enhance insulin sensitivity. Transplantation of WAT into these mice resulted in complete restoration of FGF21 responsiveness, confirming that adipose tissue is the site that plays a major role in the antidiabetic activity of FGF21 (141).

FGF21 has demonstrated the ability to augment pancreatic insulin levels and safeguard pancreatic islets from glucolipotoxicity and cytokine-induced apoptosis (163). AMPK signaling is crucial in regulating pancreatic β-cell activity and apoptosis; its activation and transduction inhibit β-cell apoptosis and diminish lipid buildup (93), which is likely to be an effect of FGF21 activation of the AMPK signaling pathway. It was found that AMPK signaling is necessary for the adult β-cell phenotype during weaning and that AMPK prevents β-cell maturation and provides an aberrant phenotype by inhibiting mTORC1 signaling. In addition, downregulation of AMPK leads to mitochondrial degeneration, which can indirectly contribute to the development of diabetes, so AMPK signaling is the first step in β-cell maturation and normal function (164, 165). AMPK signaling has been found to improve β-cell function as well as protect β-cells from apoptosis in several drug experiments, including Rhodiola rosea glycoside, mulberry pigment, and kaempferol (166–168). AMPK and SIRT1 interact with each other to regulate the action of SIRT1, which activates AMPK signaling to augment fatty acid oxidation, hence facilitating β-cell recovery and endocrine progenitor development, while also contributing to the regulation of β-cell function and survival (1, 169). The activation of AMPK and SIRT1 is linked to various positive outcomes in the maintenance of glucose homeostasis in insulin-resistant conditions (170).

FGF21 plays a significant role in lipid metabolism by promoting WAT browning, fat oxidation, and tissue thermogenesis to reduce body weight, triglycerides, and cholesterol. And lipotoxicity is also an important factor leading to IR. Lipotoxicity not only leads to apoptosis of pancreatic β-cells but also leads to inflammation, which in turn promotes the development of IR. Therefore, FGF21 can reduce the harm of lipotoxicity and improve IR by regulating lipid metabolism and immune homeostasis.

### 4.4 The role of FGF21 in immune homeostasis

The inflammatory response has a huge impact on IR progression, especially in insulin-target tissues such as muscle, adipose tissue, and liver. Therefore, improving inflammation is equally a therapeutic goal in the treatment of IR and T2DM (171, 172). And FGF21 can act on the immune and inflammatory systems. Research indicates that inflammation diminishes the functionality of FGF21, which notably inhibits pro-inflammatory factors, including tumor necrosis factor-α (TNF-α), interleukin-6 (IL-6), IL-1β, and monocyte chemotactic protein-1 (MCP-1) (173, 174). In a therapeutic study with FGF21 analogs, FGF21 was found to decrease pro-inflammatory marker expression and increase anti-inflammatory marker expression in non-human primates (175). FGF21 can induce a shift in macrophage phenotype from pro-inflammatory to pro-repair *in vitro (* 174). In acute pancreatitis mice, knockdown of FGF21 exacerbated inflammatory response and fibrosis, and restoration of FGF21 attenuated inflammation and fibrosis. The above findings confirm that FGF21 can improve the diabetic disease state by inhibiting inflammatory factor expression (176).

Lipotoxicity is closely related to IR and involves multiple pathways (177). Lipotoxicity arises from overnutrition, excessive fat storage, and catabolism, resulting in metabolic dysfunctions in peripheral organs, including muscle, pancreas, liver, and adipose tissue (178). When the storage capacity of adipose tissue is exceeded, free fatty acid spillover results in the formation of fatty acyl-coenzyme A, diglycerides (DAG), and ceramides in muscle tissue (179). The buildup of these harmful lipids significantly affects the onset of insulin resistance in skeletal muscle. Prolonged exposure to lipotoxicity results in the release of pro-inflammatory cytokines by adipose tissue and alters the equilibrium between inflammation and metabolism, which are significant factors in lipotoxicity. Ceramides induce cellular stress and death, impairing muscle insulin sensitivity by decreasing AKT (179, 180), and PKB activity, and serve as primary inflammatory mediators of muscle insulin resistance (181, 182). Ceramide has been shown to have a favorable correlation with the development of insulin resistance in the context of lipotoxicity (182–184). Toll-like receptor 4 (TLR4) significantly contributes to the onset of insulin resistance (IR) and inflammation, being expressed in insulin-responsive tissues. It induces IR by enhancing the transcription of pro-inflammatory genes and activating pro-inflammatory kinases, including JNK, IKK, and p38, among others. Pro-inflammatory kinases obstruct IRS phosphorylation, hinder insulin signaling, and impede insulin action (185, 186). These kinases additionally promote NF-κB signaling, resulting in heightened inflammatory responses (185, 187, 188). NF-κB binds to the promoter region of the solute carrier family 2 member 4 (Slc2a4) gene, thereby downregulating GLUT4 expression transcription (189, 190). FGF21 inhibits pro-inflammatory factor activity and NF-kB signaling, preventing inflammation and hepatic fibrosis (191), this may come from the activation of AMPK signaling (192). AMPK is an inhibitor of acute pro-inflammatory responses, and salicin, olive bitter moss, and naringenin all ameliorate oxidative stress or inflammation-mediated insulin resistance by stimulating the activation of AMPK signaling (93). AMPK response activity decreases with age (193), which also results in the disruption of FGF21-mediated AMPK signaling.

### 4.5 The role of FGF21 in redox homeostasis

Oxidative stress is also a cause of insulin resistance (IR), and FGF21 can reduce oxidative stress (191, 194–196). FGF21 mitigates oxidative damage and cytotoxicity through its influence on MAPK and JNK pathways (196). Chronic mitochondrial stress correlates with the activation of response pathways, including the mitochondrial unfolded protein response (UPRmt) and integrated stress response (ISR), in FGF21 target tissues such as the liver, adipose tissue, and skeletal muscle (197, 198). The IRS promotes the phosphorylation of eukaryotic initiation factor 2α (eIF2α) and activates ATF4. Following IRS activation, there is a promotion of mitochondrial UPRmt activation, which mitigates mitochondrial dysfunction (199).ATF4 mediates the transcription of FGF21 (200–203), thereby regulating systemic metabolic homeostasis (140, 204). In murine models of mitochondrial stress, the upregulation of FGF21 serves as a protective mechanism against mitochondrial damage and metabolic disorders. In models exhibiting impaired mitophagy, there is an upregulation of FGF21 expression, which facilitates thermogenesis in adipose tissue (161). The targeted deletion of autophagy-related gene 7 (ATG7) in skeletal muscle inhibits mitophagy and induces FGF21 secretion, thereby preventing insulin resistance (38). FGF21 serves as a crucial metabolic mediator, enhancing mitochondrial function while decreasing inflammation and apoptosis in skeletal muscle (205).

Notably, while chronic mild mitochondrial stress can trigger adaptive responses and improve metabolism, prolonged and sustained mitochondrial stress can lead to metabolic disorders with IR, T2DM, and obesity (206, 207). During oxidative phosphorylation, mitochondria produce reactive oxygen species (ROS) as toxic byproducts. When the production of ROS exceeds the scavenging capacity, redox imbalance occurs (208, 209), damaging mitochondrial structure, cellular DNA and proteins, etc., leading to mitochondrial dysfunction (210). Mitochondrial dysfunction correlates with insulin resistance in skeletal muscle, liver, and adipose tissue (211–213). Compromised mitochondrial oxidative capability, increased ROS, and diminished ATP generation rates all contribute to the onset of insulin resistance (206), while also impacting adipose tissue function and disturbing adipose tissue homeostasis (207, 213, 214). Excessive production of ROS also stimulates the JNK, IKK, and NF-κB pathways, disrupting cellular homeostasis (215). Oxidative stress arises from excessive ROS production in mitochondria, resulting in endoplasmic reticulum stress and activating the UPR. This ROS-induced oxidative stress exacerbates further oxidative stress, creating a detrimental cycle that damages cellular components and induces transcriptional alterations in insulin resistance (216).In addition, β-cell death and oxidative stress are likewise closely linked, and overproduction of ROS leads to oxidative damage in β-cells. Oxidative stress induces FOXO1 expression, leading to PDX1 nuclear translocation and stimulating β-cell dysfunction (93). FGF21 has been shown to mitigate endoplasmic reticulum stress (149, 217, 218), drug-induced endoplasmic reticulum stress, and adipose degeneration due to endoplasmic reticulum stress through MAPK (219, 220). Furthermore, the treatment of FGF21 enhanced mitochondrial function in hepatocytes during murine tests (78, 221), and the absence of FGF21 resulted in the buildup of hepatic reactive oxygen species, which was mitigated by FGF21 supplementation (194). At the same time, FGF21 induces activation of pathways that help protect cells from oxidative stress and inhibit pro-cell death pathways. Physiologically, FGF21 oxidizes stress by upregulating Nrf2-mediated antioxidant capacity (222). FGF21 has been demonstrated to safeguard mice from acetaminophen-induced hepatotoxicity by augmenting PGC-1α/Nrf2-mediated antioxidant capacity in the liver, and it also protects mouse liver from D-galactose-induced oxidative stress in hepatocytes by improving Nrf2-mediated antioxidant capacity (194, 222). Thus, FGF21 achieves treatment of IR in response to oxidative stress by improving mitochondrial function, decreasing ROS production, activating AMPK to inhibit inflammatory responses, and promoting insulin signaling.

In summary, the improvement and treatment of IR by FGF21 is multifaceted, including improving the damaged insulin signaling pathway to enhance insulin signaling, promoting the production and release of biological factors such as lipocalin to enhance insulin sensitivity in adipose tissue, regulating lipid metabolism to reduce lipotoxicity, and attenuating inflammation and oxidative stress. At the same time, the multiple effects of FGF21 and CNS coordinate with each other to regulate body metabolism and reverse insulin resistance.

FGF21 emerges as a pleiotropic metabolic regulator that orchestrates systemic glucose and lipid homeostasis through interconnected pathways. Its actions span the central nervous system (CNS), peripheral tissues, and immune-metabolic networks, collectively mitigating insulin resistance via three key mechanisms: (1) Neuroendocrine Regulation: CNS-specific FGF21 signaling modulates dietary preferences, thermogenesis, and hepatic insulin sensitivity by activating hypothalamic nuclei (e.g., PVN, SCN) and suppressing glucocorticoid-driven lipolysis. (2) Peripheral Metabolic Reprogramming: FGF21 enhances insulin-dependent glucose uptake in skeletal muscle and liver while driving insulin-independent glucose disposal via GLUT1 upregulation and adipose tissue browning. Notably, its synergistic effects with lipocalin amplify lipid oxidation and mitigate lipotoxicity in adipocytes and hepatocytes. (3) Anti-Inflammatory and Antioxidant Defense: By suppressing NF-κB/JNK pathways and activating AMPK/SIRT1, FGF21 reduces pro-inflammatory cytokine production, protects pancreatic β-cells from glucolipotoxicity, and restores mitochondrial function impaired by oxidative stress.

The therapeutic potential of FGF21 is underscored by its ability to simultaneously target multiple IR drivers—lipotoxicity, inflammation, and oxidative damage—while compensating for adipose tissue dysfunction. However, species-specific variations in receptor sensitivity (e.g., human vs. rodent KLB expression) and pharmacokinetic limitations (e.g., short half-life in primates) necessitate further optimization of analogs for clinical translation. Future research should prioritize elucidating tissue-specific FGF21 responses and developing delivery systems to enhance its metabolic benefits while minimizing off-target effects.

## 5 FGF21 analogs

Natural FGF21 has a short half-life of 30 min-120 min, which depends on the species (142, 223). FGF21 is vulnerable to cleavage of its structural domain by the endopeptidase fibroblast activating protein (FAP), and the removal of 10 residues at the C-terminus, specifically Pro-171 and Ser-172, diminishes FGF21’s affinity for the co-receptor KLB (14, 224, 225). Therefore, the development of FGF21 analogs is crucial (**Table 1**). However, it is worth noting that different organisms have different sensitivities to the effects of FGF21. For example, in individuals with obesity and type 2 diabetes, FGF21 analogs attenuate dyslipidemia but have less impact on glycemic control, which demonstrates the differences that still exist among different FGF21 analogs and the differences in action between species (72).

**Table 1:** Comparison table of key characteristics of FGF21 analogs.

FGF21 analogs	Potency (relative to FGF21)	Half-life	The main side effects	Remarks
PEG-FGF21	~10-20times	Extension (specific data varies with PEGylation degree)	Mild gastrointestinal reactions (e.g., nausea) Potential immunogenicity (due to PEG modification)	PEGylation extends the half-life and improves pharmacokinetics
FGF21 fusion protein	~5-10times	Significantly extended (e.g., by several days)	Injection site reactions May increase the risk of thrombosis	Fusion with antibodies or albumin for half-life extension
FGF21 Allosteric Modulator	~2-5times	Short (requires frequent dosing)	Elevated liver enzymes	Small-molecule compounds targeting the FGFR-KLB complex
			Potential metabolic disorder	
GLP-1/FGF21 dual agonist	Synergistic Effect (1 + 1>2)	Moderate (partially dependent on GLP-1)	Gastrointestinal reactions (nausea/vomiting)	The satiety effect of GLP-1 combined with the metabolic regulation of FGF21
			Hypoglycemia risk (monitoring required)	
Antibody-conjugated FGF21	~15-30times	Extremely long (several weeks)	High immunogenicity	Antibody-mediated delivery of FGF21 to specific tissues (e.g., the liver)
			Off-target toxicity (e.g., hepatotoxicity)	

Currently, research on FGF21 analogs is still in the experimental stage, for example, LY2405319, a variant with FGF21 proto-life created in Lilly’s lab, reduces aggregation in solution by introducing additional disulfide bonds through the Leu118Cys and Ala134Cys mutations and reduces aggregation in the solution by deletion of the four amino-terminal amino acid residues His-Pro-Ile- Pro97 to avoid protein hydrolytic cleavage (72). Its effects on both insulin and lipids were positive in diabetic subjects, but glucose uptake was not as effective as in monkey subjects (154, 226, 227). PF-05231023, a long-acting FGF21 analog, is a combination of the protein with the antibody scaffold CovX-2000, intended for weekly injection, exhibiting superior pharmacokinetics in human kinetics and safety (228, 229), and capable of reducing blood glucose and cholesterol levels in murine models (230, 231). BMS-986036, the third medication, is derived from polyethylene glycol-modified recombinant human FGF2. Clinical trials indicate that it demonstrates favorable safety and tolerability characteristics while considerably decreasing the incidence of NASH in obese and diabetic patients (228, 229, 232, 233). Nonetheless, the medication exhibits restricted efficacy against human adipose tissue-FGFR1c (234), potentially resulting in diminished insulin sensitization of adipose tissue. BIO98-100, a recent FGF21 analog (235), is a novel glycopolyglycolated variant that interacts comparably with human FGF21 and FGFR1c to elicit a following. This analog enhanced fasting glucose, cholesterol, and triglyceride levels in diabetic monkeys (235). The analog possesses a half-life of 55–100 hours in humans, enhancing its previously limited half-life, and research suggests it enhances insulin sensitivity, lipocalin levels, and triglyceride concentrations. A highly anticipated contemporary analog is the fusion protein of FGF21 with human immunoglobulin 1Fc, which enhances affinity for KLB and possesses a half-life of 3-3.5 days, referred to as AKR-001 (236, 237). In phases 1 and 2 of clinical trials, this analog enhanced insulin sensitivity and lipoprotein levels while simultaneously inhibiting adipose tissue catabolism and hepatic neoadipogenesis, demonstrating good tolerability (234, 236). The cumulative effects of most FGF21 analogs are substantial in enhancing lipid metabolism, although they demonstrate less efficacy in ameliorating glycemic alterations in humans compared to animal trials (154, 230, 235). However, this does not mean that FGF21 is not helpful for diabetes and insulin resistance in humans, and FGF21 analogs can improve the insulin signaling pathway by improving lipid metabolism and reducing lipotoxicity and chronic inflammation associated with obesity in humans. Moreover, since FGF21 works differently in different organisms, and perhaps analogs that do not work well in animal studies are effective in humans, there are still many FGF21 analogs worth developing in humans!

## 6 Safety of FGF21

Several studies of FGF21 are still progressing, and the safety of its clinical production has attracted attention. FGF21 has been suggested to have potential effects on bone turnover as it has been observed in animal models that FGF21 stimulates adipogenesis in the bone marrow, leading to bone loss (238). Bone loss was observed in mice treated with pharmacological doses of FGF21 (100). Nonetheless, this was not observed in another same experiment involving mice (239). Also in rodents, FGF21 increases thermogenesis and promotes adrenergic receptor activation in adipose tissue by spiking the sympathetic nerves of the BAT, which may lead to symptoms triggered by sympathetic hyperexcitability, such as hyperhidrosis, but is rarely seen in human studies using FGF21 analogs. These occurrences may be due to differences in FGF21 analogs or interspecies differences that lead to different effects.

The most common adverse reactions in FGF21 analog trials are gastrointestinal reactions, including diarrhea and nausea. However, this adverse reaction is usually transient, similar to metformin, which occurs mainly at the beginning of treatment and can be tolerated after a period of use (234, 240, 241). here have also been cases of FGF21 analogs causing elevated heart rate and blood pressure with use, but this is an isolated case (230). It is worth mentioning that in rodents and monkeys, administration of FGF21 drug therapy does not cause adverse effects on mitosis or hypoglycemia. FGF21 has been shown to influence female reproduction (242, 243), as seen by infertility in mice that overexpress this factor. This may pertain to energy insufficiency resulting from chronic energy depletion, which can be ameliorated when FGF21 transgenic sterile mice are on a high-fat diet (243). However, further research is needed to determine whether the administration of FGF21 treatment affects female fertility.

## 7 The future and prospects of FGF21

This article has systematically elucidated the signaling pathways of FGF21 and its mechanisms of action. However, several critical scientific questions remain unresolved. While FGF21 has been demonstrated to play a pivotal role in glucose and lipid metabolism as well as immune homeostasis in coordination with the central nervous system, the precise molecular mechanisms underlying its organ-specific effects remain poorly understood. Although accumulating evidence highlights FGF21’s immense therapeutic potential for metabolic disorders such as obesity, insulin resistance, and type 2 diabetes, key gaps persist in our understanding of its systemic actions.

Preclinical studies have shown that antidiabetic drugs like metformin and thiazolidinediones (TZDs) not only upregulate FGF21 expression but also activate AMPK while improving mitochondrial function - effects that synergize with FGF21’s metabolic regulatory properties. Nevertheless, significant challenges remain in drug development:

First, the pharmacological efficacy of FGF21 analogs exhibits remarkable species-specific differences. Analogues showing remarkable efficacy in rodent models often demonstrate substantially diminished effects in non-human primates or human trials, suggesting complex inter-species variations in FGF21 signaling. The molecular basis for these discrepancies remains to be fully characterized.

Second, while animal studies consistently demonstrate FGF21’s beneficial effects on glycemic control, human clinical trial results show considerable heterogeneity. This discrepancy underscores the limitations of current experimental models (e.g., diet-induced obesity mouse models) in fully replicating the pathophysiology of human metabolic diseases. There is an urgent need to develop more physiologically relevant animal models or organoid systems that better mimic human disease conditions.

Safety concerns represent another major hurdle for FGF21 analog development. Current data indicate these analogs may cause mild gastrointestinal side effects, alter lipid profiles, and potentially induce hepatotoxicity, though comprehensive long-term safety data remain limited. Particularly concerning are reports of dose-dependent immunogenic responses in some clinical trials, which could compromise drug efficacy through neutralizing antibody production. Moreover, the pharmacokinetic profiles of engineered FGF21 analogs differ substantially from endogenous FGF21, raising questions about their impact on physiological homeostasis.

Based on these challenges, we propose the following priority areas for future research:

1. Organ-Specific Mechanistic Studies

- Employ tissue-specific gene knockout models and single-cell sequencing technologies to delineate FGF21’s differential effects across organs (liver, adipose tissue, skeletal muscle, brain).
- Investigate the crosstalk mechanisms between peripheral tissues and the central nervous system in FGF21-mediated metabolic regulation.

2. Cross-Species Pharmacology Research

- Conduct comparative genomic and proteomic analyses to identify species-specific determinants of FGF21 signaling efficacy.
- Develop improved preclinical evaluation systems incorporating multiple species data

3. Next-Generation Drug Delivery Systems

- Design targeted nanocarrier platforms for tissue-specific FGF21 delivery (e.g., liver-targeted nanoparticles).
- Explore sustained-release formulations to optimize pharmacokinetic profiles.

4. Long-Term Safety Assessment

- Implement large-scale, long-term clinical trials monitoring immunological, cardiovascular, and hepatic safety parameters.
- Establish biomarkers for early detection of adverse immune responses.

5. Combination Therapy Strategies

- Investigate synergistic mechanisms between FGF21 analogs and existing antidiabetic agents (GLP-1 receptor agonists, SGLT2 inhibitors).
- Develop optimized combination regimens for metabolic syndrome management.

Addressing these challenges through multidisciplinary approaches will be essential to translate FGF21-based therapies from bench to bedside, ultimately realizing their potential to revolutionize treatment paradigms for metabolic diseases.
